# Dr. James O. Emmett

**Published:** 1914-06

**Authors:** 


Dr. James O. Emmett, New York Homoeopathic Medical College, 1867; died suddenly at his home in Fonthill, Ont., near Welland, April 30, aged 71. He had practiced in Fonthill throughout his active life.

